# Clinical Features, Molecular Biology, and the Metastatic Microenvironment in Lung Cancer Brain Metastases: Implications for Treatment Decisions

**DOI:** 10.1002/advs.202502626

**Published:** 2025-07-28

**Authors:** Yixiang Zhu, Danming He, Zan Hou, Mei Lan, Ye Zhang, Qifeng Wang

**Affiliations:** ^1^ Department of Radiation Oncology Sichuan Clinical Research Center for Cancer Sichuan Cancer Hospital & Institute Sichuan Cancer Center Affiliated Cancer Hospital of University of Electronic Science and Technology of China Chengdu Sichuan 610041 China; ^2^ State Key Laboratory of Molecular Oncology Department of Medical Oncology National Cancer Center/National Clinical Research Center for Cancer/Cancer Hospital Chinese Academy of Medical Sciences & Peking Union Medical College 17 Pan‐jia‐yuan South Lane, Chaoyang District Beijing 100021 China; ^3^ Department of Radiation Oncology National Cancer Center/National Clinical Research Center for Cancer/Cancer Hospital Chinese Academy of Medical Sciences and Peking Union Medical College Beijing 100021 China

**Keywords:** lung cancer, brain metastases, clinical characteristics, metastasis factors, treatment advances

## Abstract

Brain metastases (BM) represent a highly aggressive, clinically distinct subtype of lung cancer, often associated with poor prognosis. Historically, treatment options for BM have been limited and largely nonspecific. However, recent advancements in clinical and preclinical research have led to substantial improvements in patient outcomes. This review focuses on BM arising from lung cancer, providing an overview of recent findings related to clinical characteristics, diagnostic strategies, and early metastatic processes. Multi‐omics analyses have elucidated the molecular mechanisms underlying tumor initiation and progression, with particular emphasis on the role of the tumor microenvironment. In this review, preclinical BM models, detailed signaling pathways, and emerging clinical therapies are also discussed. Current treatment approaches are multidisciplinary, and multi‐omics technologies enhance both diagnosis and therapeutic strategies by revealing the complex biology of BM. Continued research is needed to identify BM‐specific drug targets, particularly those involved in crossing the blood–brain barrier and remodeling the brain microenvironment. Addressing these challenges is crucial for improving clinical outcomes in patients with BM.

## Graphical abstract

Brain metastases (BM) from lung cancer are aggressive with poor prognosis. This review covers clinical features, diagnosis, early metastasis, and multi‐omics‐elucidated mechanisms, especially the tumor microenvironment of lung cancer with BM. It also discusses preclinical models, signaling pathways, and emerging therapies. Capturing the information is essential for improving clinical outcomes in patients with BM. More research is needed for BM‐specific targets to boost outcomes.

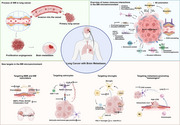

## 1 Introduction

The brain is one of the most common sites for metastasis from lung cancer, and patients with brain metastases (BM) have a poorer prognosis than those without BM^[^ ^1^ ^]^ Lung cancer is pathologically classified into: non‐small cell lung cancer (NSCLC), accounting for 80–85% of cases, and small cell lung cancer (SCLC), accounting for 15–20% of cases.^[^ ^2^ ^]^ Among patients with non‐metastatic NSCLC, the risk of developing BM is 11% for lung adenocarcinoma, 6% for squamous cell carcinoma (SCC), and 12% for large cell carcinoma; in SCLC, BM is present at the initial diagnosis in ≈10% of patients, affecting 40–50% within two years.^[^ ^3^ ^]^

The “seed and soil” hypothesis posits that BM arise from interactions between disseminated cancer cells and the brain microenvironment. The brain's distinctive microenvironment encompasses resident cell populations, unique structural composition (blood–brain barrier [BBB]), and specialized immune processes,^[^ ^4^, ^5^ ^]^ requiring cancer cells seeding into the brain to possess the capacity not only to cross the BBB but also to survive and proliferate within the metastatic niche. The acquisition of distinct driver mutations and metabolic reprogramming is critical for BM tumor cells to adapt to the brain microenvironment. A comprehensive understanding of the complex processes and biological mechanisms underlying NSCLC BM, as well as the unique characteristics of the immune microenvironment in BM, is essential for the development of effective, targeted therapeutic strategies. These emerging insights must be translated into innovative treatments that can be integrated into evolving conventional therapeutic paradigms.

Current treatment modalities for BM include surgical resection, radiotherapy, targeted therapy, immunotherapy, or combinations thereof. Targeted therapies, such as tyrosine kinase inhibitors (TKIs) directed against epidermal growth factor receptor (EGFR) and anaplastic lymphoma kinase (ALK), have led to major therapeutic advances and prolonged survival. However, their efficacy in patients with BM is limited. BM is the leading cause of treatment failure with first‐ and second‐generation EGFR‐TKIs and first‐generation ALK‐TKIs.^[^ ^6^, ^7^ ^]^ Although third‐generation EGFR‐TKIs and second‐ and third‐generation ALK‐TKIs provide improved intracranial control, long‐term survival remains poor owing to intracranial progression and a lack of effective therapeutic options.^[^ ^8^, ^9^, ^10^ ^]^ Immune checkpoint inhibitors have improved outcomes in patients with NSCLC lacking driver mutations; however, intracranial response rates remain low, at ≈16.4%.^[^ ^11^, ^12^ ^]^ In SCLC, BM treatment still relies on conventional approaches, including radiotherapy and chemotherapy, with limited benefit observed from targeted therapies or immune checkpoint inhibitors. Therefore, elucidating the mechanisms underlying BM development is essential for the design of effective therapeutic strategies. Recent advances have enhanced our understanding of the biological processes that drive BM occurrence. This review systematically highlights the latest developments in lung cancer with BM, summarizing the clinical features, diagnosis, underlying molecular mechanisms, and emerging therapeutic strategies. Our aim is to provide researchers with a comprehensive overview and deeper insights into this complex and evolving field.

## 2 Clinical Features of BM

### 2.1 Non‐squamous NSCLC (ns‐NSCLC) with BM

With advances in targeted therapy, lung cancer management has entered the era of molecular diagnostics and precision treatment. EGFR mutations are most common in Asian populations, with a prevalence of up to 50%.^[^ ^13^ ^]^ Hsu et al.^[^ ^14^ ^]^ reported that the incidence of BM was similar between patients with EGFR mutations and those with wild‐type EGFR (30.6% vs 25.1%, p = 0.36) in newly diagnosed stage IV NSCLC. However, cumulative BM rates at 1 and 3 years were significantly higher in patients with EGFR mutations than in those with wild‐type EGFR at 39.0% versus 28.1% (p = 0.041) and 39.2% versus 28.2% (p = 0.038), respectively. Similarly, Wang et al.^[^ ^15^ ^]^ reported an initial BM rate of 26% in patients with EGFR‐mutated advanced ns‐NSCLC and 24.6% in those with wild‐type EGFR. Cumulative 1‐ and 3‐year BM rates were higher in the EGFR‐mutated group (35.3% vs 29.7% and 46.2% vs 35.8%, respectively; p = 0.008). No significant difference in BM incidence has been reported between the EGFR exon 19 deletion and the L858R mutation.^[^ ^16^, ^17^ ^]^

Gillespie et al.^[^ ^18^ ^]^ reviewed BM incidence across NSCLC genotypes, reporting an overall rate of 28.6% (95% CI 26.1–31.0), with the highest rates observed in ALK‐positive (34.9%) and RET‐rearranged (32.2%) tumors. The annual BM incidence rates were as follows: wild‐type, 0.13 (95% CI 0.11–0.16); EGFR‐mutant, 0.16 (95% CI 0.11–0.21); ALK‐positive, 0.17 (95% CI 0.10–0.27); KRAS‐mutant, 0.10 (95% CI 0.06–0.17); ROS1‐rearranged, 0.13 (95% CI 0.06–0.28); and RET‐rearranged NSCLC, 0.12 (95% CI 0.08–0.17).

Baek et al.^[^ ^19^ ^]^ reported that the median interval from diagnosis to BM development in patients with stage IV NSCLC (predominantly ns‐NSCLC, 84.6%) did not significantly differ between EGFR‐mutant and wild‐type groups (13.4 vs 8.8 months, p = 0.229). Eichler et al.^[^ ^20^ ^]^ similarly reported no significant difference (19.0 vs 14.0 months; p = 0.27). Takano et al.^[^ ^16^ ^]^ observed that BM in both EGFR‐mutant and wild‐type adenocarcinomas more frequently involved the frontal, parietal, cerebellar, and occipital lobes. However, BM associated with the EGFR L858R mutation often involves the caudate, cerebellum, and temporal lobes and are located closer to the brain surface than those with exon 19 deletion (p = 0.003) or wild‐type EGFR (p<0.001), with median distances of 12.4 mm (10.9–13.9 mm) versus 15.5 mm (14.4–16.7 mm) and 12.4 mm (10.9–13.9 mm) versus 16.1 mm (15.2–17.1 mm), respectively.

Sekine et al.^[^ ^21^ ^]^ reported that patients with EGFR mutations were more likely to develop multiple BM than those with wild‐type EGFR. The median number of BM was 5 (range, 1–100) for the exon 19 mutation and 4 (range, 1–20) for the exon 21 mutation, compared with 3 (range, 1–38) in wild‐type EGFR (p = 0.024). Takano et al.^[^ ^16^ ^]^ reported mean BM diameters of 4.8 mm (3.9–6.4 mm) and 5.2 mm (3.9–8.8 mm) for exon 19 and 21 mutations, respectively, which is comparable to that in wild‐type EGFR (5.6 mm, 3.6–8.7 mm). Sekine et al.^[^ ^21^ ^]^ also noted less BM in patients with exon 19 (7.1 mm, 3.0–2.2 mm) and 21 (10 mm, 3.2–41.0 mm) mutations and less peritumoral edema (0.3 mm [0.0–36.0 mm] and 6.1 mm [0.0–57.0 mm], respectively). Conversely, the median diameters of BM and peritumoral edema in patients with wild‐type EGFR were larger at 11.0 mm (2.9–62.0 mm; p = 0.0016) and 8.8 mm (0.00–76.6 mm; p = 0.0036), respectively. The proportions of BM with peritumoral edema <10 mm, <20 mm, and <30 mm were higher in the exon 19 group (73.5%, 91.2%, and 97.1%, respectively), than in exon 21 (42.9%, 78.6%, and 85.7%, respectively) or wild‐type EGFR groups (37.0%, 50.3%, and 61.1%, respectively).

### 2.2 SCC with BM

Among 26430 patients with BM, Cagney et al.^[^ ^22^ ^]^ reported a 5.2% incidence of BM at diagnosis in patients with SCC. Lee et al.^[^ ^23^ ^]^ observed a similar rate (5.0%) among 564 patients with lung SCC. Goncalves et al.^[^ ^3^ ^]^ indicated a 6.0% incidence of BM in patients with non‐metastatic SCC. For patients with BM from lung cancer, the reported BM rate in squamous NSCLC is 13.5%.^[^ ^16^ ^]^ BMs in SCC typically occur in the frontal, parietal, cerebellar, and occipital lobes, with a mean lesion depth of 14.2 mm (8.8–24.2 mm; p = 0.43).^[^ ^16^ ^]^ Single and multiple metastases were equally distributed (50.0% each).^[^ ^22^ ^]^

### 2.3 SCLC with BM

Seute et al.^[^ ^24^ ^]^ found that 10% of patients with SCLC had BM at initial diagnosis, increasing to 50% within 2 years. Cagney et al.^[^ ^22^ ^]^ reported a 15.8% BM incidence at diagnosis in patients with SCLC. Goncalves et al.^[^ ^3^ ^]^ observed a similar rate (18.0%) in patients with non‐metastatic SCLC. The BM rate and mean depth were 14% and 14.1 mm (8.5–24.5), respectively. As with other subtypes, BMs in SCLC most commonly involve the frontal, parietal, cerebellar, and occipital lobes.^[^ ^16^ ^]^ Chaung et al.^[^ ^25^ ^]^ reported that the median number and size of the largest BM in SCLC were 3 and 20 mm, respectively. Komatsu et al.^[^ ^26^ ^]^ found consistent results, with the most frequent number of BM being 1–3, tumor size being 355 mm^2^, and maximum tumor diameter being 20 mm.

These studies suggest that lung cancer subtypes exhibit distinct characteristics of BM. Ns‐NSCLC is associated with a higher initial incidence of BM compared with SCC and SCLC. Although SCLC demonstrates a rising cumulative incidence of BM over 2–3 years, this trend supports the potential benefit of prophylactic cranial irradiation in this subgroup. Notably, EGFR mutation status is not significantly associated with the time to BM development or the site of BM occurrence. However, patients with EGFR‐mutant tumors tend to develop a higher number of BM and smaller lesion size, possibly reflecting increased metastatic dissemination efficiency. Further studies are warranted to clarify the associations among specific oncogenic drivers, the anatomical distribution, and biological characteristics of BM (**Table** **1**).

**Table 1:** Clinical features of BM in patients with lung cancer.

Type		Incidence rate of initial diagnosis	3‐year cumulative incidence rate	Time to BM (median)	Most common site	Number of BM (median)	Tumor size (median)
Ns‐NSCLC	EGFR wild type	24.6–25.1%	28.2–39.2%	8.8–14.0 months	frontal, parietal	2–3	11.0 mm
	EGFR mutation	26.0–30.6%	39.2–46.2%	13.4–19.0 months	frontal, parietal	4–5	4.8–7.1 mm
SCC		5.0–13.5%	–	–	frontal, parietal	1	14.2 mm
SCLC		10.0–18.0%	50%^*^	–	frontal, parietal	3	14.1–20 mm

BM, brain metastases; Ns‐NSCLC, non‐squamous non‐small cell lung cancer; SCC, squamous carcinoma; SCLC, small cell lung cancer; EGFR, epidermal growth factor receptor. ^*^2‐year cumulative incidence rate of BM.

### 2.4 Diagnosis of BM

Radiomics has become a widely used method for screening and treatment assessment in patients with BM.^[^ ^27^ ^]^ Recent advances in imaging technologies and molecular diagnostics have substantially enhanced both the detection and management of BM. Liquid biopsy has emerged as a critical tool in BM management, contributing to diagnosis, prognostic stratification, treatment response prediction, and detection of tumor progression.^[^ ^28^ ^]^

### 2.5 Radiomics and BM

Advances in medical imaging and artificial intelligence (AI) have significantly improved the diagnostic accuracy of BM and facilitated a more precise prediction of treatment responses. Gong et al.^[^ ^29^ ^]^ analyzed 602 patients with stage IIIA–IVB NSCLC and extracted 1106 radiomic features from pretreatment CT scans, demonstrating that combining radiomic and clinical data improved BM risk prediction (p<0.05). Zhu et al.^[^ ^30^ ^]^ trained 3D neural networks on ^18^F‐FDG PET/CT images to predict BM, integrating shallow lung features and deep lung‐brain features. These features demonstrated high predictive value (p<0.001, coefficient >0.8). Yichu et al.^[^ ^31^ ^]^ extracted radiomic features from CT images of primary lung lesions and applied machine learning models (logistic regression, support vector machines [SVM], k‐nearest neighbors, random forests) to predict BM in newly diagnosed lung cancers. The optimal SVM model, integrating both intratumoral and peritumoral features, was validated as a predictive imaging biomarker. Multitask deep learning networks have also been employed to predict molecular profiles, such as *EGFR* mutation status, in BM versus primary lung tumors.^[^ ^32^ ^]^ Models targeting *EGFR* 19Del/21L858R mutations and wild‐type have achieved area under the curve (AUC) >0.97; however, caution is warranted to mitigate overfitting and adversarial model behavior in clinical AI applications.^[^ ^33^ ^]^

Additionally, radiogenomics has improved the characterization of tumor heterogeneity and prediction of treatment responses, including targeted therapy and immunotherapy. Deng et al.^[^ ^34^ ^]^ used radiomic features from MRI and RNA sequencing of tumor specimens in a multicenter NSCLC cohort to construct a survival prediction model (AUC >0.8), which was independent of clinical variables (hazard ratio [HR] = 31.3). Low‐risk radiomic features correlated with CD8^+^T cell infiltration and interferon pathway activation. Zhou et al.^[^ ^35^ ^]^ applied a multimodal framework integrating clinical, histopathological, neuroimaging, and multi‐omics data, which achieved 87% accuracy in predicting BM progression, surpassing the performance of four board‐certified pathologists (57.3%). These findings suggest that multimodal models incorporating clinical, histopathological, neuroimaging, and genomic data may offer a promising approach for predicting therapeutic efficacy in patients with NSCLC and BM. However, the diagnostic and predictive accuracy of such models may be limited by sample sizes, risks of overfitting, and challenges in interpretability. Thus, further efforts are required to enhance the integration of medical and engineering disciplines.

### 2.6 Liquid Biopsy

Liquid biopsy plays a critical role in the management of patients with BM, offering valuable applications in tumor detection, prognostic assessment, evaluation of treatment response, and monitoring of disease progression. This minimally invasive approach involves analysis of biomarkers, such as circulating tumor DNA (ctDNA), circulating tumor cells (CTCs) (see *Initiating Metastasis Factors*), and non‐coding RNAs.

### 2.7 ctDNA

ctDNA consists of DNA fragments released into the bloodstream by tumor cells.^[^ ^36^ ^]^ These fragments carry tumor‐specific genetic and epigenetic alterations. Through real‐time dynamic monitoring, ctDNA serves as a non‐invasive biomarker to track tumor evolution, assess treatment responses, and identify resistance mechanisms. Alder et al.^[^ ^37^ ^]^ analyzed serum ctDNA from 253 patients with BM and found that EGFR alterations were most frequently detected in patients with NSCLC BM (p<0.001) and in those with isolated BM with stable extracranial disease (p = 0.08). Furthermore, Ryu et al.^[^ ^38^ ^]^ found that detectable plasma ctDNA before treatment was associated with an increased risk of new metastases, especially in the brain; shorter tumor doubling time (p = 0.039); and reduced overall survival (OS) (p = 0.019). Zhang et al.^[^ ^39^ ^]^ reported that changes in ctDNA allele frequency preceded or coincided with clinical disease progression, allowing early detection of drug resistance and relapse in NSCLC and meningeal metastasis. However, because of the BBB, plasma is not an ideal source to evaluate the genetic characteristics and prognosis of BM.^[^ ^40^ ^]^ Aldea et al.^[^ ^41^ ^]^ reported that ctDNA was detectable in 52% of patients with isolated intracranial progression, compared with 84% in those with extracranial progression and 92% in those with intracranial and extracranial progression (p<0.00001). Detection rates of driver mutations were lower in intracranial lesions in patients with isolated progression (37% vs 77% and 73%, respectively), as were resistance‐associated genomic alterations (6% vs 45% and 44%, respectively).

Unlike plasma ctDNA, cerebrospinal fluid (CSF) ctDNA exhibits higher sensitivity for detecting BM. In patients with lung cancer and BM, the most frequently altered genes in the CSF were *EGFR* (84.0%), *TP53* (60.6%), *MET* (24.5%), *CDKN2A* (23.4%), *MYC* (21.3%), *NTRK*1 (20.2%), and *CDK6* (16.0%).^[^ ^42^ ^]^ Zheng et al.^[^ ^43^ ^]^ reported similar results, with enrichment of *MYC, CDKN2A, STK11, NTRK1, TP53, SMAD4, RB1, CDK6, CDK4*, and *MET* in CSF ctDNA. Wu et al.^[^ ^44^ ^]^ found that CSF ctDNA identified all BM mutations in 83.33% of patients, whereas plasma ctDNA detected only 27.78%. CSF ctDNA showed higher concordance with BM than did plasma ctDNA (99.33% vs 67.44%). Moreover, Zheng et al.^[^ ^43^ ^]^ demonstrated that CSF ctDNA reflects the BM tumor burden, with the sum of the longest diameters being significantly greater in the ctDNA‐positive patients than in those who were ctDNA‐negative. Additionally, the mutation allele frequency of CSF ctDNA was strongly correlated with BM tumor size (r = 0.95).^[^ ^44^ ^]^ In patients with NSCLC and meningeal metastasis, CSF ctDNA demonstrated greater sensitivity than cytology (p<0.001).^[^ ^39^ ^]^ A recent meta‐analysis has shown that the diagnostic power of ctDNA from CSF is better than that from the plasma (RR = 1.46, 95% CI 0.93–2.29; p<0.01).^[^ ^45^ ^]^ Furthermore, CSF ctDNA positivity can independently predict poor OS in NSCLC and BM (HR = 1.87, 95% CI 1.48–2.38, p<0.001).^[^ ^44^ ^]^ CSF ctDNA‐guided treatment strategies may improve survival and help overcome therapeutic resistance.^[^ ^43^ ^]^ Overall, CSF ctDNA demonstrates superior performance to plasma ctDNA in accurately diagnosing BM and informing risk stratification. Longitudinal monitoring may further enhance the clinical utility of CSF ctDNA‐guided interventions.

### 2.8 Non‐coding RNAs (ncRNAs)

ncRNAs are categorized by length, function, and structure into groups, including microRNAs (miRNAs), long ncRNAs (lncRNAs), and circular RNAs (circRNAs). These ncRNAs have demonstrated potential in liquid biopsy applications. Wei et al.^[^ ^46^ ^]^ identified that miR‐550a‐3‐5p was enriched in exosomes from patients with lung cancer and BM. Additionally, miR‐522‐3p was elevated in serum samples from this population.^[^ ^47^ ^]^ Liu et al.^[^ ^47^ ^]^ further showed that miR‐522‐3p modulates BBB permeability by altering tight junction proteins, thereby facilitating BM development. Wu et al.^[^ ^48^ ^]^ reported that lnc‐MMP2‐2 functions as a competing endogenous RNA for miR‐1207‐5p, resulting in depression of EPB41L5 and disruption of tight junctions, which in turn increases BBB permeability and promotes lung cancer BM. Although circRNAs have emerged as potential diagnostic and prognostic biomarkers—and even therapeutic targets—in breast cancer BM,^[^ ^49^, ^50^ ^]^ their role in lung cancer BM remains unclear. Taken together, although current findings highlight the potential of ncRNAs to influence lung cancer BM, their diagnostic utility requires further investigation.

## 3 Initiating Metastasis Factors

### 3.1 Genetic Characteristics

In 1889, Paget^[^ ^51^ ^]^ proposed the “seed and soil” theory of metastasis, positing that specific tumor cells (“seeds”) preferentially colonize certain organs (“soil”) with supportive microenvironments. Private genomic alterations may confer brain‐tropic properties to tumor cells. Nguyen et al.^[^ ^52^ ^]^ employed MSK‐IMPACT to sequence 15632 primary and 10143 metastatic tumor samples across 50 tumor types and 21 metastatic sites, revealing distinct metastatic patterns. For instance, lung adenocarcinoma more frequently metastasized to the brain—but less often to the liver (22% vs 42%)—than did neuroendocrine carcinoma (34% vs 19%). Compared with primary tumors, metastatic lesions exhibited greater chromosomal instability, genomic alterations, and mutation burden. Mutations in *TP53* and *EGFR* and amplification of TERT were more frequent in BM from lung adenocarcinoma, whereas *RBM10* mutations were less common. Jiang et al.,^[^ ^53^ ^]^ using whole‐exome sequencing of 12 matched lung primary tumors and BM, found that only 10.9% (402/3703) of BM mutations were present in primary tumors. *EGFR* (50.0%) and *TP53* (33.3%) mutations that predominated in BM were shared with primary tumors, with *EGFR* and *TP53* co‐altered in 66.7% of *EGFR*‐mutant cases. Several pathways were enriched in BM, including Rap1 signaling (q = 0.024), tight junctions (q = 5.63×10^−8^), actin cytoskeleton regulation (q = 7.23×10^−11^), calcium signaling (q = 0.045), ABC transporters (q = 0.013), and central carbon metabolism (q = 5.01×10^−6^). Shih et al.^[^ ^54^ ^]^ sequenced BM from 73 patients with lung adenocarcinoma and compared them with 503 primary lung cancer tissue samples from The Cancer Genome Atlas. BM samples more frequently harbored *MYC* (12% vs 6%), *YAP1* (7% vs 0.8%), *MMP13* (10% vs 0.6%), and *CDKN2A/B* deletions (27% vs 13%). Overexpression of these genes increased BM incidence in xenograft models. Further studies are needed to determine whether these alterations are essential for BM development and whether targeting them could reduce BM risk.

### 3.2 CTCs

CTCs shed from primary or metastatic lesions into the bloodstream or lymphatic system^[^ ^55^ ^]^ and are key initiators of metastasis. Once in circulation, CTCs may exist as single cells or multicellular clusters,^[^ ^56^ ^]^ and successful metastasis depends on their interaction with permissive distant microenvironments.^[^ ^57^ ^]^

CTC clusters are typically heterogeneous,^[^ ^58^ ^]^ and single‐cell sequencing has revealed the heterogeneity among CTCs.^[^ ^59^, ^60^ ^]^ In patients with SCLC, Stewart et al.^[^ ^61^ ^]^ reported variable epithelial‐mesenchymal transition (EMT) scores among CTCs, indicating transcriptional diversity. Sun et al.^[^ ^62^ ^]^ showed spatial heterogeneity of CTCs from hepatocellular carcinoma across four vascular sites. Tumor subtype and vascular origin influence organ‐specific metastatic potential.^[^ ^62^, ^63^ ^]^ CTCs exhibit “pre‐set” organotropism and display distinct molecular traits depending on their source. Wurth et al.^[^ ^64^ ^]^ used organoid models of CTCs from patients with breast cancer and found, via single‐cell transcriptomics, that high HER3 activity may promote lung‐specific metastasis. The brain's restrictive microenvironment imposes unique selective pressures on metastatic CTCs.

Klotz et al.^[^ ^65^ ^]^ identified Semaphorin‐4D expression in CTCs from patients with metastatic breast cancer. Its interaction with Plexin‐B1, highly expressed in brain microvascular endothelial cells, facilitated CTC passage across the BBB. CTC‐expressed MYC also upregulated GPX1, reducing oxidative stress from microglial activation and enhancing BM survival. Gonzalez et al.^[^ ^66^ ^]^ performed single‐cell RNA sequencing on 15 BM samples from various cancers and found that genes involved in dormancy exit—*MYC, DNMT1, MEST, and PEG10—*were upregulated, whereas genes involved in inflammation and stress responses were downregulated. Chen et al.^[^ ^67^ ^]^ demonstrated that *RAC1*, a gene enriched in metastatic versus primary lung adenocarcinoma tissue, promoted BM rather than liver, bone, or adrenal metastases. *RAC1* enhanced cell proliferation, invasiveness, and migration, while its inhibition attenuated these traits. Fu et al.^[^ ^68^ ^]^ reported higher bacterial loads in breast tumor tissues than in adjacent normal tissues, with even higher loads in CTC clusters and lung metastases. These bacteria modulate the actin cytoskeleton to help CTCs resist mechanical stress in circulation and enhance migration to metastatic sites.

### 3.3 Exosomes

**Figure 1:**
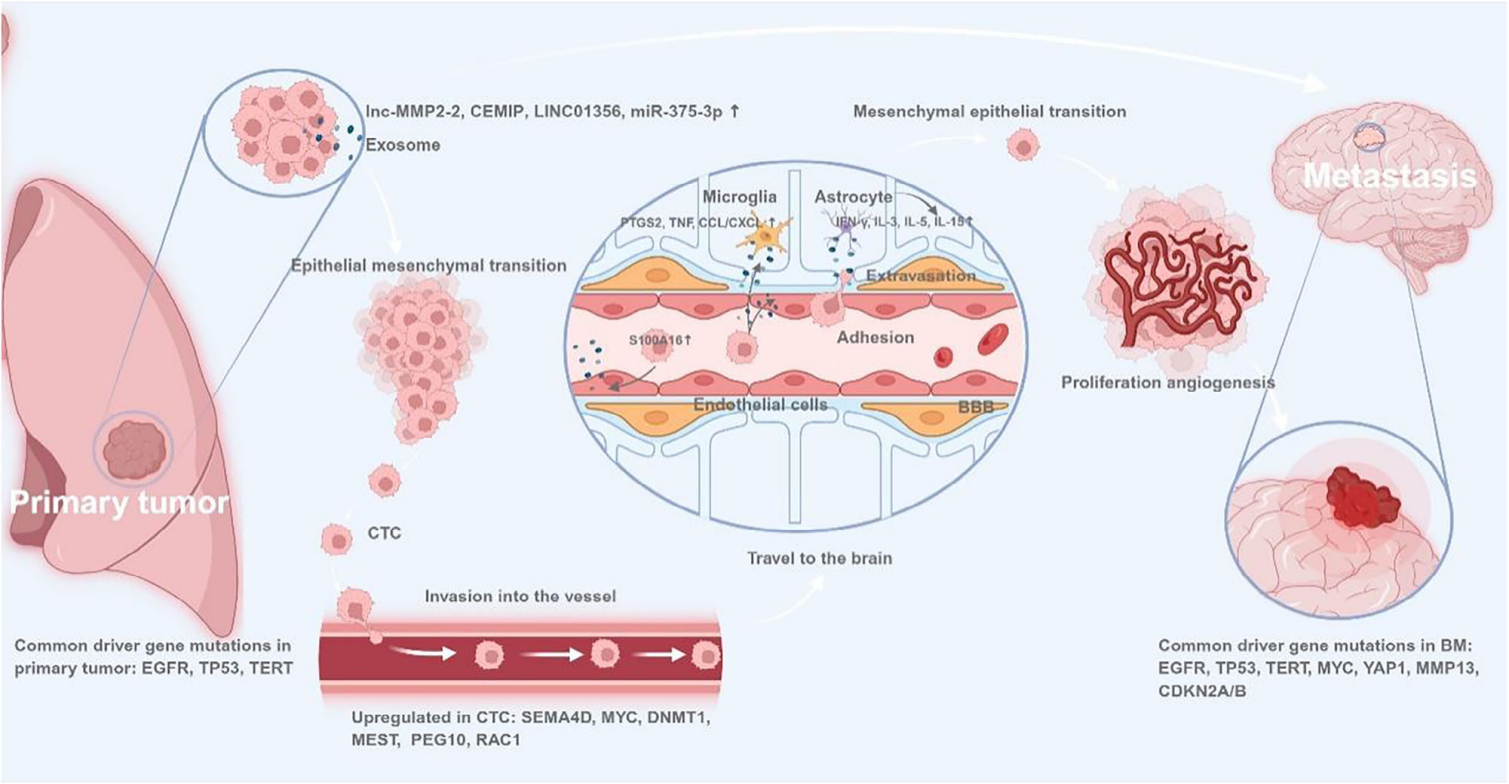
Process of BM in lung cancer. BM is a multistep process involving: 1) local invasion and basement membrane degradation; 2) intravasation of tumor cells into blood or lymphatic vessels; 3) entry of CTCs into systemic circulation; 4) arrest and extravasation of CTCs from blood or lymphatic vessels into the brain; and 5) colonization and proliferation to form BM lesions. Throughout this process, tumor cells can remodel the microenvironment to support their survival and dissemination via autocrine and paracrine signaling. BM: brain metastases, CTC: circulating tumor cells, BBB: blood–brain barrier.

Exosomes are extracellular vesicles that mediate intercellular communication and influence key processes in cancer metastasis. Xu et al.^[^ ^69^ ^]^ reported that co‐culturing brain microvascular endothelial cells with SCLC cells significantly upregulated S100A16 expression in SCLC cells, and inhibition of exosome release in endothelial cells blocked this effect. Elevated S100A16 enhanced tumor cell survival by modulating mitochondrial function within the brain. Moreover, accumulating evidence implicates exosomes in promoting metastasis via EMT‐related pathways.^[^ ^70^, ^71^ ^]^ Wu et al.^[^ ^72^ ^]^ demonstrated that TGF‐β1‐mediated exosomal lnc‐MMP2‐2 facilitates BM in NSCLC by enhancing BBB permeability through the miR‐1207‐5p/EPB41L5 axis. Similar findings were observed for exosomal LINC01356 and miR‐375‐3p, which also promoted lung cancer BM by increasing BBB permeability.^[^ ^73^, ^74^ ^]^ Exosomes also modulate the metastatic microenvironment. Rodrigues et al.^[^ ^75^ ^]^ isolated tumor‐derived exosomes from mice with BM and identified elevated levels of the cell migration‐inducing and hyaluronan‐binding protein in brain‐tropic, but not lung‐ or bone‐tropic, cells. These exosomes are absorbed by brain endothelial cells and microglia, upregulating proinflammatory genes (*PTGS2, TNF, CCL/CXCL*), remodeling the vasculature, and promoting BM. Tumor‐derived exosomes critically mediate premetastatic niche formation by triggering astrocytic secretion of inflammatory cytokines (IFN‐γ, IL‐3, IL‐5, IL‐15) that establish microenvironments, sustaining tumor survival through chronic inflammation while evading immune detection via immunosuppressive reprogramming^[^ ^76^ ^]^ (**Figure** **1**).

## 4 The Metastatic Microenvironment

The brain microenvironment, often termed the “soil” for metastasis, is unique due to its specialized cell populations, including astrocytes, microglia, and neurons, which present therapeutic challenges.^[^ ^77^ ^]^ This niche exhibits an immunosuppressive and fibrotic landscape, with infiltrates of cancer‐associated fibroblasts (CAFs), microglia, astrocytes, neutrophils, B cells, T cells, myeloid cells, and neurons. The roles of intracranial microenvironmental cells and components in the process of BM are described below, with a focus on their interactions with tumor cells.

### 4.1 CAFs

CAFs are pivotal components of the tumor stroma. Within the tumor microenvironment (TME), they remodel the extracellular matrix (ECM), altering its mechanical properties and influencing both cancer and immune cell behavior. CAFs also promote angiogenesis and exhibit strong immunomodulatory functions, facilitating immune evasion.^[^ ^78^, ^79^, ^80^ ^]^ You et al.^[^ ^81^ ^]^ identified CAF subpopulations in the lung cancer BM microenvironment. Intercellular communication analyses revealed that myo‐CAFs interact with vascular endothelial cells to promote angiogenesis and with macrophages to induce their differentiation into the SPP1 phenotype. Fibrosis is a hallmark feature of the BM microenvironment.^[^ ^82^ ^]^ Using spatial transcriptomics, Zhang et al.^[^ ^4^ ^]^ analyzed 44 samples from patients with NSCLC and brain tissue samples from 7 patients without BM and observed that BM showed upregulation of fibrosis‐related genes (collagen, fibronectin, vimentin), whereas neuronal and glial markers were downregulated. Moreover, immunosuppression persisted regardless of fibrotic status.^[^ ^4^ ^]^ Compared to non‐metastatic brain tissue, peritumoral BM regions exhibited increased angiogenesis and reduced fibrosis and neural function. Non‐fibrotic peritumoral areas expressed high levels of inflammatory cytokines and chemokines (e.g., TNF, IL1B, and CXCL10), whereas fibrotic zones exhibited elevated M2 markers (CD163, TGFB1).

### 4.2 Microglia

Microglia are the resident macrophages of the central nervous system (CNS) and are central regulators of the brain's immune microenvironment. They help repair BBB damage induced by CTCs and contribute to BM development after systemic therapy.^[^ ^83^ ^]^ Once metastases are established, microglia lose their phagocytic function and shift toward tumor growth promotion by increasing the release of anti‐inflammatory cytokines, recruiting peripheral monocytes, and inhibiting T‐cell proliferation, thereby fostering an immunosuppressive milieu. In the early metastatic stages, endothelial‐derived DKK1 induced by lung cancer‐derived exosomes drives a pro‐tumorigenic microglial phenotype, supporting premetastatic niche formation.^[^ ^84^ ^]^

### 4.3 Astrocytes

Astrocytes, the most abundant non‐neuronal cells in the brain, become reactive upon stimulation and are the first responders to invading tumor cells. Initially, they resist metastasis by producing plasminogen activators, but tumor‐secreted serpins neutralize this response, supporting tumor survival.^[^ ^85^ ^]^ Zhang et al.^[^ ^86^ ^]^ showed that astrocyte‐derived exosomes containing miR‐19A downregulate PTEN in tumor cells, increasing CCL2 expression and recruiting peripheral myeloid cells, thereby supporting tumor growth in the brain. Zhang et al.^[^ ^4^ ^]^ revealed that astrocytes are reprogrammed into immature and proinflammatory states in BM.

### 4.4 Neutrophils

Neutrophils, comprising 50–70% of circulating myeloid‐derived leukocytes, are central to innate immunity. In cancer, neutrophils accumulate not only in primary tumors but also in distant organs.^[^ ^87^ ^]^ In BM, neutrophils exhibit heightened immune and inflammatory signaling, including TNFα signaling, but diminished reactive oxygen species production.^[^ ^88^ ^]^ During the early phases of BM development, primary tumors trigger astrocyte‐mediated inflammation, recruiting neutrophils to the metastatic niche.^[^ ^89^ ^]^ Their phenotypic plasticity and role in metastasis remain subjects of ongoing intensive research.

### 4.5 B cells

B cells are crucial mediators of humoral immunity, exerting anti‐tumor effects via antibody‐dependent cytotoxicity and complement activation.^[^ ^90^ ^]^ However, their role in BM remains insufficiently characterized. Generally, B‐cell infiltration is markedly lower than T‐cell infiltration across different cancer types, regardless of tissue origin.^[^ ^91^ ^]^ Wang et al.^[^ ^92^ ^]^ performed single‐cell sequencing of primary lung tumors, matched BM tissues, and reported that although B cells were less enriched in BM, they exhibited notable clonal expansion, suggesting a potential role in immune evasion in the metastatic setting.

### 4.6 T cells

T cells represent one of the most abundant immune cell populations in lung cancer BM, alongside monocyte‐derived macrophages and neutrophils, collectively accounting for over 35% of the TME. This suggests a dominant role for T cells in shaping the immune milieu. Jiang et al.^[^ ^53^ ^]^ compared 12 paired samples of primary lung adenocarcinoma and BM and found a significantly immunosuppressive microenvironment in BM, with elevated CD8^+^ (p = 0.048) and CD4^+^Foxp3^+^ (p = 0.036) T cell infiltration, and programmed cell death protein 1 (PD‐1) expression (p = 0.047). Similarly, Gonzalez et al.^[^ ^66^ ^]^ conducted single‐cell RNA sequencing of 15 BM specimens from lung, breast, melanoma, and ovarian cancers. Among all immune cells, T cells exhibited the greatest heterogeneity, frequently exhibiting characteristics of exhaustion and functional inactivity. Tagore et al.^[^ ^5^ ^]^ conducted single‐cell RNA sequencing, T‐cell receptor sequencing, single‐cell spatial sequencing, and whole‐genome sequencing on BM and primary tumors from treatment‐naive patients with NSCLC. BM exhibited fewer CD8^+^ T cells but a higher prevalence of Tregs, naive NK cells, and naïve T cells.

### 4.7 Myeloid Cells

Predominant myeloid populations in BM include tumor‐associated macrophages (APOE^+^/C1QB^+^/TREM2^+^ macrophage‐associated myeloid cells [MAMs]) and myeloid‐derived suppressor cells (IL1B^+^/FCN1^+^/S100A8^+^ MAMs). Effector memory CD8^+^ and CD4^+^ T cells expressed PD‐1 and programmed death‐ligand 1 (PD‐L1), with an inverse association observed between tumor cell proliferation and immune cell infiltration. These findings underscore the immunosuppressive nature of the BM microenvironment, which is orchestrated in part by myeloid cells.^[^ ^66^, ^93^ ^]^ For example, loss of CXCL3 in myeloid cells upregulates CXCL10, promoting the recruitment of VISTA^+^/PD‐L1^+^ CNS‐resident myeloid cells to the leptomeningeal compartment, thereby facilitating immune invasion in leptomeningeal carcinomatosis.^[^ ^93^ ^]^ In addition, macrophages in BM demonstrated elevated CD163 protein expression relative to matched alveolar macrophages.^[^ ^5^ ^]^

### 4.8 Neurons

Neuron‐tumor interactions play a critical role in BM progression. Deshpande et al.^[^ ^94^ ^]^ demonstrated that co‐culture of neurons with BM‐derived breast cancer cells enhanced tumor‐neuron communication, reduced EMT, and upregulated synaptic mediators, promoting tumor adaptation and colonization in the brain. Sanchez‐Aguilera et al.^[^ ^95^ ^]^ proposed that BM‐induced alterations in neural activity are not attributable to mass effect, but instead to molecular changes in neuronal communication. Biermann et al.^[^ ^96^ ^]^ observed marked differences in intracranial and extracranial metastatic tumors, identifying a subset of neuro‐related genes likely involved in brain tropism. Collectively, these studies underscore that neuronal interactions not only influence tumor cell invasion but may also contribute to their successful engraftment and growth within the brain.

### 4.9 ECM

The ECM is a non‐cellular structural component of the TME, comprising a network of fibrous proteins, such as collagens, glycoproteins, and proteoglycans.^[^ ^97^ ^]^ The brain ECM is structurally and compositionally distinct. As in other organs, the brain ECM forms a basement membrane around the vasculature; however, the parenchymal ECM surrounding neural and glial cells is enriched in hyaluronan and proteoglycans. Gan et al.^[^ ^98^ ^]^ reported that high ECM gene expression in patients with breast cancer was associated with a higher risk of BM and recurrence, indicating a strong link between ECM components and BM development.

### 4.10 BBB

**Figure 2:**
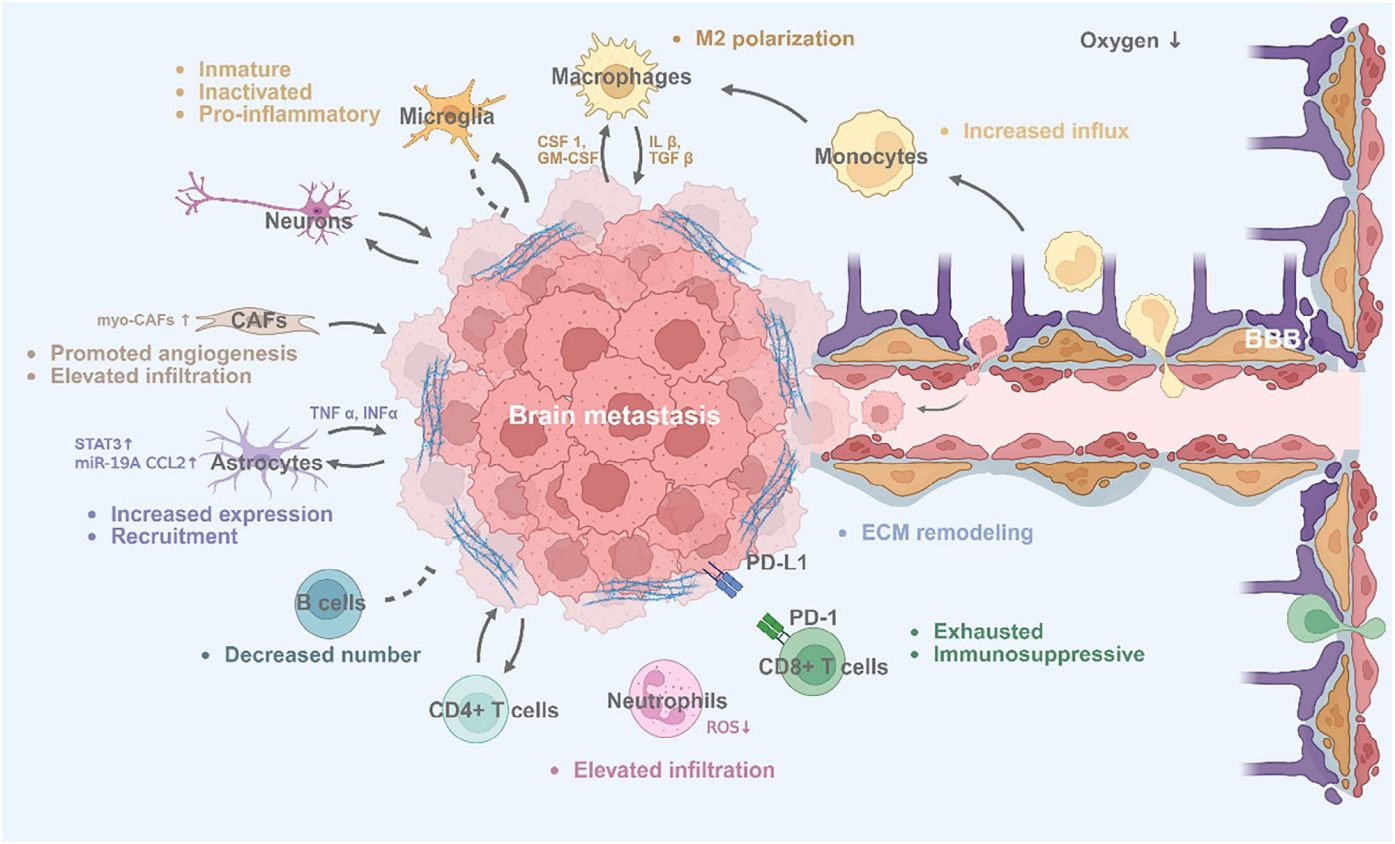
Overview of tumor–immune interactions in the BM microenvironment. This schematic illustrates established interactions between immune cell subsets and metastatic tumor cells, providing a conceptual overview of immune dynamics in the BM. Arrows indicate supportive interactions; inhibitory arcs represent suppression; dotted lines denote interactions supported by limited evidence. Both myeloid and lymphoid immune cell populations exhibit dynamic crosstalk with tumor cells in the metastatic microenvironment. BM: brain metastases, BBB: blood–brain barrier, ECM: extracellular matrix.

The BBB—composed of capillary endothelial cells with tight junctions, basement membranes, and astrocyte dendrites—is the first structure encountered by tumor cells during BM formation.^[^ ^99^ ^]^ Under physiological conditions, the BBB preserves CNS homeostasis by restricting the entry of drugs, toxins, ions, and other foreign substances.^[^ ^100^ ^]^ Tight junctions are fundamental for BBB integrity.^[^ ^101^ ^]^ Vascular endothelial cells, a core BBB component, interact with metastatic tumor cells via adhesion molecules. In early‐stage BM from NSCLC, tumor cells adhere to endothelial cells via VLA‐4/VCAM‐1, ALCAM/ALCAM, and LFA‐1/ICAM‐1 interactions^[^ ^102^ ^]^ (**Figure** **2**).

In summary, comprehensive single‐cell and spatial analyses of the TME in BM enhance our understanding of cellular dynamics and intercellular interactions, thereby elucidating the immune landscape of the BM microenvironment. These findings collectively indicate that the cellular composition of BM differs markedly from that of primary tumors and is essential for identifying potential diagnostic and therapeutic targets for BM.

## 5 Preclinical Models for BM

BM exhibits substantial intratumoral heterogeneity in genomic alterations that influence immune infiltration and disease progression.^[^ ^103^ ^]^ The lack of preclinical models that recapitulate this heterogeneity remains a major barrier to translational research.

Cancer cell lines are the most widely used models for studying metastasis.^[^ ^104^ ^]^ Common approaches for establishing BM models include intracranial,^[^ ^105^, ^106^ ^]^ intracardiac,^[^ ^50^ ^]^ and intracarotid^[^ ^107^ ^]^ injections. Intracranial injections allow direct tumor implantation into the brain parenchyma and rapid lesion formation but lack adequate interaction with the brain microenvironment. Moreover, intracranial injections disrupt the BBB, fail to replicate the metastatic cascade, and do not allow for the evaluation of drug permeability.^[^ ^108^, ^109^ ^]^ In contrast, intracardiac and intracarotid injections preserve BBB integrity and can objectively evaluate drug efficacy post‐BBB traversal.^[^ ^108^ ^]^ However, these systemic models are technically complex, may produce extracranial tumors, and often fail to represent primary tumor escape mechanisms, increasing animal disease burden.^[^ ^110^, ^111^ ^]^

In vitro culture of cancer cells alters gene expression, epigenetic profiles, and cellular metabolism, often eliminating in vivo heterogeneity.^[^ ^112^ ^]^ Compared with cell line models, genetically engineered mouse models (GEMMs) better mimic human tumor biology but are limited by slow metastasis development and low BM incidence.^[^ ^113^ ^]^ Patient‐derived xenograft (PDX) models preserve genomic complexity, and some develop spontaneous metastases that reflect clinical patterns.^[^ ^114^, ^115^ ^]^ Techniques such as intracardiac, intracranial, and intracarotid injections are currently being investigated for establishing BM PDX models.^[^ ^116^, ^117^ ^]^ Despite their advantages, PDX models are associated with low metastatic potential, high cost, and limited utility in assessing BBB permeability. However, they may provide valuable insights into second‐line drug sensitivity in relapsed or metastatic cases.^[^ ^118^ ^]^

Organoid‐derived models are 3D cultures that preserve patient‐specific genetic and phenotypic features and are increasingly used in drug screening. These models are expandable, can be cryopreserved, and retain long‐term stability.^[^ ^119^ ^]^ Qu et al.^[^ ^120^ ^]^ studied interactions between SCLC and astrocytes using assembloids composed of SCLC aggregates and cortical organoids. Organoid‐based BM models overcome several limitations of cancer cell lines, GEMMs, and PDXs, and they offer translational potential. Choe et al.^[^ ^121^ ^]^ developed a 3D in vitro model using tumor cells and brain organoids derived from human embryonic stem cells. Understanding the biological underpinnings and BM TME is critical for advancing organoid models. Although technically demanding and costly, these models offer promise for future preclinical research. Additionally, Li et al.^[^ ^122^ ^]^ developed a physiologically based pharmacokinetic model using a four‐compartment permeability framework to predict drug distribution in plasma and the CNS.

Microfluidic chips are a robust technological platform offering distinct advantages, including the ability to replicate the in vivo microenvironment, low sample consumption, high levels of automation, and seamless integration. These systems play a pivotal role in cancer research by enabling the establishment of metastasis cascade models.^[^ ^123^, ^124^ ^]^ Kim et al.^[^ ^125^ ^]^ developed a 3D microfluidic platform that mimics the complex brain TME in BM from NSCLC, using patient‐derived tumor cells, astrocytes, and brain endothelial cells. This system provides a promising drug screening platform for precision therapy and preclinical evaluation of treatments targeting metastatic and drug‐resistant tumors. Liu et al.^[^ ^124^ ^]^ constructed a multi‐organ biomimetic microfluidic chip comprising two organ‐on‐a‐chip units: an upstream “lung” unit and a downstream “brain” unit. The chip features a functional “BBB” structure and recapitulates key pathophysiological processes of BM.

In summary, both in vitro and in vivo models aim to better recapitulate the pathophysiological characteristics of human BM. Given the complexity of BM, selecting appropriate preclinical models is essential for advancing mechanistic understanding and therapeutic development. Therefore, developing experimental systems that are cost‐effective, allow for controlled experimental conditions, and support long‐term culture and application is of paramount importance. For drug screening purposes, selecting disease models or model combinations that align with the pharmacological characteristics of the drug and its target pathways is crucial for ensuring a reliable and comprehensive evaluation.

## 6 Treatment Advances

Although the critical vulnerabilities of BM have been increasingly elucidated through studies of their development and TME, most current clinical trials focus on systemic therapeutic effects and lack BM‐specific strategies. The development of novel therapeutic agents—such as dual‐targeting compounds and agents with enhanced BBB penetrability and site‐specific targeting—offers promising strategies to improve survival outcomes in patients with BM.

### 6.1 Targeted Therapy

**EGFR**: *The Brain* study^[^ ^126^ ^]^ was the first to compare the efficacy of the first‐generation EGFR‐TKI icotinib with whole‐brain radiation therapy (WBRT) combined with chemotherapy as first‐line treatment for patients with EGFR‐mutant NSCLC and asymptomatic BM. Icotinib significantly improved intracranial progression‐free survival (iPFS) (10.0 months vs 4.8 months, p = 0.014) and overall PFS (6.8 months vs 3.4 months, p<0.001). Considering the limited BBB permeability of first‐generation EGFR‐TKIs, Dong et al.^[^ ^127^ ^]^ compared the outcomes of EGFR‐TKIs alone versus in combination with brain radiotherapy in EGFR‐mutated NSCLC with BM. Across 12 studies (11 assessing first‐generation and 1 assessing second‐generation EGFR‐TKIs), combined therapy significantly prolonged iPFS (HR = 0.62, p<0.001) and OS (HR = 0.64, p<0.001). Similarly, Wang et al.^[^ ^128^ ^]^ reported that upfront radiotherapy plus EGFR‐TKIs yielded longer iPFS (HR = 0.72, 95% CI 0.53–0.97, p = 0.028) and OS (HR = 0.70, 95% CI 0.53–0.93, p = 0.015) than did EGFR‐TKI monotherapy, especially in patients with a limited number of BM (HR = 0.54, 95% CI 0.41–0.72, p<0.001). In contrast, Pike et al.^[^ ^129^ ^]^ assessed upfront stereotactic radiosurgery (SRS) in TKI‐naïve patients with EGFR‐ and ALK‐driven NSCLC and BM receiving CNS‐penetrant TKIs. Upfront SRS improved time‐to‐intracranial progression (HR = 0.63, 95% CI 0.42–0.96; p = 0.033) and local control (HR = 0.30, 95% CI 0.16–0.55; p<0.001), but not OS. The benefit was greater in patients with larger BM diameters (≥1 cm).

Afatinib, an oral irreversible ERBB family blocker targeting EGFR, HER2, HER3, and HER4, differs mechanistically from first‐generation EGFR‐TKIs. Schuler et al.^[^ ^130^ ^]^ compared afatinib with chemotherapy as a first‐line treatment for patients with EGFR‐mutant BM. The overall response rate (ORR) was 72.9% and 25%, and the PFS was 8.2 months and 5.4 months (p = 0.0297) for the afatinib and chemotherapy groups, respectively. No significant difference was observed in OS (22.4 months vs 25.0 months, p = 0.641). In the LUX‐LUNG‐7 study,^[^ ^131^ ^]^ afatinib with gefitinib showed no significant difference in PFS (HR = 0.76, 95% CI 0.41–1.44) or OS (HR = 1.16, 95% CI 0.61–2.21) among patients with BM. Both first‐ and second‐generation are substrates for efflux transporters, limiting BBB penetration and efficacy against NSCLC BM.

The third‐generation EGFR‐TKI osimertinib has demonstrated good intracranial efficacy. Among patients with BM and EGFR‐TKI failure secondary to T790M mutation, osimertinib achieved an intracranial ORR (iORR) of 70%, an intracranial disease control rate (iDCR) of 93%, and a median iPFS of 11.7 months.^[^ ^132^ ^]^ As a first‐line agent, osimertinib significantly improved PFS (15.2 months vs 9.6 months, p<0.001) and iPFS (not reached versus 13.9 months, p = 0.014) compared to first‐generation TKIs.^[^ ^133^ ^]^ Jänne et al.^[^ ^134^ ^]^ also reported longer iPFS (HR = 0.58, 95% CI 0.33–1.01) and higher iORR (73% vs 69%) with osimertinib plus platinum‐pemetrexed versus osimertinib alone.

Other third‐generation EGFR‐TKIs have also shown promise. Almonertinib significantly improved iPFS (29.0 months vs 8.3 months, HR = 0.30, 95% CI 0.14–0.66, p = 0.0015) over gefitinib, with similar iORR (85.7% vs 75.0%; p = 0.312).^[^ ^135^ ^]^ Furmonertinib demonstrated longer iPFS (20.8 months vs 9.8 months, HR = 0.40, 95% CI 0.23–0.71, p = 0.0011) and a higher iORR (91% vs 68%; p = 0.0277) over gefitinib.^[^ ^136^ ^]^ Lazertinib also significantly improved iPFS (28.2 months vs 8.4 months, HR = 0.42, 95% CI 0.20–0.89, p = 0.02) and iORR (94% vs 73%) compared to gefitinib.^[^ ^137^ ^]^

Tozuka et al.^[^ ^138^ ^]^ retrospectively found that upfront local therapy followed by osimertinib significantly improved OS (HR = 0.37, 95% CI 0.16–0.87) and iPFS (HR = 0.36, 95% CI 0.15–0.87) compared with osimertinib alone. Collectively, these findings suggest that both third‐generation EGFR‐TKIs and local therapies significantly enhance intracranial outcomes over first‐ and second‐generation agents.

**ALK**: In the ALESIA trial,^[^ ^139^ ^]^ alectinib, a second‐generation ALK inhibitor, was compared with crizotinib, a first‐generation ALK inhibitor, as first‐line treatment for ALK‐positive advanced NSCLC. Alectinib significantly improved iORR (94.1% vs 28.6%) in patients with BM compared to crizotinib. The ASCEND‐7 study ^[^ ^140^ ^]^ evaluated ceritinib, a second‐generation ALK inhibitor, in patients with ALK‐positive NSCLC, BM, and/or leptomeningeal metastases. ORR and iORR were 35.7% and 39.3%, respectively, among those previously treated with radiotherapy or ALK‐TKIs. Lorlatinib, a third‐generation alkaline phosphatase inhibitor, demonstrated markedly superior iORR over crizotinib (66% vs 20%) and significantly reduced intracranial progression at 12 months (3% vs 33%, HR = 0.06, 95% CI 0.02–0.18).^[^ ^141^ ^]^ It also significantly lowered the risk of intracranial progression (HR = 0.10, 95% CI 0.04–0.27) compared to crizotinib.^[^ ^142^ ^]^ NVL‐655, a highly selective ALK‐TKI, has shown clinical activity in patients with ALK‐positive NSCLC with BM and ALK resistance mutations.^[^ ^143^ ^]^

**ROS‐1**: Entrectinib—a multi‐target inhibitor of NTRK, ROS1, and ALK—achieved an iORR of 79.2% and a median iPFS of 12.0 months in a pooled analysis of patients with measurable BM.^[^ ^144^ ^]^ Repotrectinib, a new‐generation macrocyclic ROS1/NTRK‐TKI, showed intracranial activity in 89% of patients with ROS1‐positive NSCLC with measurable BM.^[^ ^145^ ^]^

**HER‐2**: Pyrotinib, a pan‐HER‐TKI, achieved an iORR and a PFS of 33.3% and 7.0 months, respectively, in patients with HER2‐mutant advanced NSCLC with BM.^[^ ^146^ ^]^

### 6.2 Immune Checkpoint Inhibitors

Immunotherapy is a key therapeutic approach for BM and has been associated with improved survival in several tumor types.^[^ ^147^ ^]^ Goldberg et al.^[^ ^11^ ^]^ evaluated pembrolizumab in untreated or progressive NSCLC with BM. Among patients with PD‐L1‐positive tumors, ORR and iORR were 18.9% and 29.7%, and median OS and iPFS were 9.9 months and 2.3 months, respectively. No responses were observed in PD‐L1‐negative tumors. Powell et al.^[^ ^148^ ^]^ analyzed KEYNOTE‐021, ‐189, and ‐407 studies, demonstrating that pembrolizumab plus chemotherapy improved median mPFS (6.9 months vs 4.1 months) and OS (18.8 months vs 7.6 months) compared to chemotherapy alone in patients with NSCLC with BM. Subgroup analysis of a first‐line regimen of camrelizumab plus chemotherapy showed improved PFS (9.7 months vs 6.7 months, HR = 0.57, 95% CI 0.29–1.11) and iPFS (12.7 months vs 9.9 months, HR = 0.45, 95% CI 0.21–0.96).^[^ ^149^ ^]^ Among patients receiving radiotherapy, carilizumab further improved PFS (1.2 months vs 6.7 months; HR = 0.42, 95% CI: 0.19–0.94), and iPFS (19.1 months vs 9.9 months; HR = 0.42, 95% CI: 0.17–1.01). The C‐Brain study confirmed that brain radiotherapy combined with camrelizumab and platinum‐doublet chemotherapy yielded a 6‐month PFS rate of 71.7% (95% CI 58.9–81.1).^[^ ^150^ ^]^

In the IMpower 133 study,^[^ ^151^ ^]^ atezolizumab plus chemotherapy did not significantly improve OS versus chemotherapy alone as first‐line treatment for extensive‐stage SCLC (ES‐SCLC) in the BM subgroup (HR = 1.07, 95% CI 0.47–2.43). Similarly, in the CASPIAN study,^[^ ^152^ ^]^ durvalumab plus chemotherapy yielded an OS comparable to chemotherapy alone in ES‐SCLC (HR = 0.69, 95% CI 0.35–1.31), but it delayed intracranial progression and the need for brain radiotherapy (19.2 months vs 10.2 months, HR = 0.69, 95% CI 0.50–0.95). The benmelstobart monoclonal antibody is a domestically developed PD‐L1 monoclonal antibody. The ETR701 trial^[^ ^153^ ^]^ showed that benmelstobart combined with anlotinib, etoposide, and carboplatin, prolonged OS overall, but not in the BM subgroup. Serplulimab, a domestic PD‐1 inhibitor, also failed to significantly improve OS in patients with BM compared with chemotherapy alone in the ASTRUM‐005 study^[^ ^154^ ^]^ (HR = 0.61, 95% CI, 0.32–1.13).

### 6.3 Antibody‐Drug Conjugates (ADCs)

**HER‐2**: Trastuzumab deruxtecan (T‐DXd) is an ADC that targets HER2. Li et al.^[^ ^155^ ^]^ evaluated T‐DXd in patients with HER2‐expressing NSCLC with BM. At doses of 5.4 and 6.4 mg kg^−1^, the iORRs were 50% and 30%, and the iDCRs were 92.9% and 73.3%, with median intracranial duration of response (iDORs) of 9.5 and 4.4 months, respectively.

**HER‐3**: Patritumab deruxtecan (HER3‐DXd) targets HER3. In the HERTHENA‐Lung01 study, Johnson et al.^[^ ^156^ ^]^ reported iORR and iDCR of 20% and 80%, respectively, in pretreated patients with EGFR‐mutant NSCLC with BM. The median iDOR was 9.2 months.

**DLL3**: Rovalpituzumab tesirine (Rova‐T), the first DLL3‐targeting ADC, was compared with topotecan as second‐line therapy for DLL3‐high metastatic SCLC in the TAHOE study.^[^ ^157^ ^]^ Among patients with baseline BM, OS was similar between the two groups (7.3 months vs 8.8 months; HR = 1.28, 95% CI 0.96–1.71). The MERU study^[^ ^158^ ^]^ explored Rova‐T as maintenance therapy following first‐line platinum‐based chemotherapy in extensive‐stage SCLC. Rova‐T did not improve OS (10.2 months vs 10.7 months, HR = 0.8; 95% CI 0.5–1.3) in patients with BM.

**TROP2**: Datopotamab deruxtecan (Dato‐DXd) targets TROP2. In the TROPION‐Lung05 study,^[^ ^159^ ^]^ 53 patients with pretreated advanced or metastatic NSCLC and stable BM received Dato‐DXd. ORR and DCR were 28% and 72%, respectively, with a median PFS of 5.4 months. Among 18 evaluable patients with BM, iORR and iDCR were 22% and 72%, respectively, with a median iDOR of 5.5 months (**Table** **2**).

**Table 2:** Therapies for Lung Cancer with BM.

Trial Name/Identifiers	Study Design	Treatment Arm	Inclusion of population	Control Arm	Administration (Treatment vs Control Arm)	iORR	miPFS	mOS	AEs (≥3 grade)	Drop‐out rate due to SAEs	BM failure rate
**EGFR**											
Brain (NCT01724801)^[^ ^126^ ^]^	Phase III, randomized	Icotinib	Untreated EGFR‐mutant NSCLC	WBRT + chemotherapy	Daily oral vs. Intravenously every three weeks	–	10.0 months vs. 4.8 months	–	8% vs. 38%	0% vs. 20%	12% vs. 23%
LUX‐Lung 3, LUX‐Lung 6 (NCT00949650, NCT01121393)^[^ ^167^ ^]^	Phase III, randomized	Afatinib	Untreated EGFR‐mutant NSCLC	Chemotherapy	Daily oral vs. Intravenously every three weeks	72.9% vs. 25%	8.2 months vs. 5.4 months	22.4months vs. 25.0 months	–	7.9% vs. 11.7%	–
AURA3 (NCT02151981)^[^ ^132^ ^]^	Phase III, randomized	Osimertinib	First‐line EGFR‐TKI failure and T790M‐positive NSCLC	Platinum + pemetrexed	Daily oral vs. Intravenously every three weeks	–	8.5 months vs. 4.2 months	–	23% vs. 47%	–	–
FLAURA (NCT02296125)^[^ ^6^ ^]^	Phase III, randomized	Osimertinib	Untreated EGFR‐mutant NSCLC	First‐generation TKIs	Daily oral vs. Daily oral	76.0% vs. 86.0%	not reached vs. 13.9 months	–	42% vs. 47%	15% vs. 18%	20% vs. 39%
FLAURA2 (NCT04035486)^[^ ^134^ ^]^	Phase III, randomized	Osimertinib + platinum‐pemetrexed	Untreated EGFR‐mutant NSCLC	Osimertinib	Daily oral + Intravenously every three weeks vs. Daily oral	73.0% vs. 69.0%	24.9 months v 13.8 months	–	64% vs. 29%	45% vs. 6%	24% vs. 30%
ANEAS (NCT03849768)^[^ ^135^ ^]^	Phase III, randomized	Almonertinib	Untreated EGFR‐mutant NSCLC	Gefitinib	Daily oral vs. Daily oral	85.7% vs. 75.0%	29.0 months vs. 8.3 months	–	31.4% vs. 36.4%	2% vs. 1.8%	21.6% vs. 39.7%
FURLONG (NCT04870190, NCT05379803)^[^ ^136^ ^]^	Phase III, randomized	Furmonertinib	Untreated EGFR‐mutant NSCLC	Gefitinib	Daily oral vs. Daily oral	91.0% vs. 68.0%	20.8 months vs. 9.8 months	–	32% vs. 32%	6% vs. 0	0 vs. 11%
LASER301 (NCT04248829)^[^ ^137^ ^]^	Phase III, randomized	Lazertinib	Untreated EGFR‐mutant NSCLC	Gefitinib	Daily oral vs. Daily oral	94.0% vs. 73.0%	28.2 months vs. 8.4 months	–	42% vs. 49%	13% vs. 10%	22% vs. 37%
Wang et al.^[^ ^128^ ^]^	Retrospective	Up‐front radiotherapy and EGFR‐TKI	EGFR‐mutated NSCLC	EGFR‐TKI	Daily oral + RT vs. Daily oral	–	HR = 0.72, 95%CI: 0.53‐0.97	HR = 0.70, 95%CI 0.53‐0.93	–	–	–
(NCT05184712)^[^ ^160^ ^]^	Phase III, randomized	Ivonescimab +chemotherapy	First‐line EGFR‐TKI failure NSCLC	Chemotherapy	Intravenously every three weeks vs. Intravenously every three weeks	–	5.8 months vs. 4.1 months	–	61.5% vs. 49.1%	5.6% vs. 2.5%	–
**ALK**											
ALESIA (NCT02838420)^[^ ^168^, ^169^ ^]^	Phase III, randomized	Alectinib	Untreated ALK positive NSCLC	Crizotinib	Daily oral vs. Daily oral	94.1% vs. 28.6%	–	–	41% vs. 50%^*)^	0 vs. 0^*)^	18.8% vs. 56.9%
ASCEND‐7 (NCT02336451)^[^ ^140^ ^]^	Phase II	Ceritinib	ALK positive NSCLC	–	Daily oral	12.5‐51.5%	–	–	58.3‐88.9%	4.8‐33.3%	25‐41.7%
CROWM (NCT03052608)^[^ ^141^ ^]^	Phase III, randomized	Lorlatinib	Untreated ALK positive NSCLC	Crizotinib	Daily oral vs. Daily oral	66% vs. 20%	–	–	72% vs. 56% ^*)^	21% vs. 15%^*)^	3% vs. 33%
TURBO‐NSCLC^[^ ^129^ ^]^	Retrospective	TKI	TKI‐naïve patients with EGFR‐ and ALK‐driven NSCLC with BM	TKI+SRS	Daily oral + RT vs. Daily oral	–	–	41 months vs. 40months	–	–	34% vs. 21%
**ROS‐1**											
ALKA‐372‐001, STARTRK‐1, STARTRK‐2 (NCT02097810, NCT02568267)^[^ ^144^ ^]^	Phase I/ II	Entrectinib	ROS1 fusion‐positive NSCLC	–	Daily oral	79.2%	12.0 months	–	11.0%^*)^	–	67.4%
(NCT03093116) ^[^ ^145^ ^]^	Phase I/ II	Repotrectinib	ROS1 fusion‐Positive NSCLC	–	Daily oral	89.0%	–	–	51%^*)^	7%^*)^	0 (ROS1 TKI‐naïve) and 23% (One ROS1 TKI‐pretreated and chemotherapy‐naïve)
HER‐2											
(NCT03574402)^[^ ^146^ ^]^	Phase II	Pyrotinib	HER2‐mutant NSCLC	–	Daily oral	33.3%	7.0 months	–	10.7% ^*)^	0^*)^	22.2%
Immune Checkpoint Inhibitors											
(NCT02085070)^[^ ^170^ ^]^	Phase II	Pembrolizumab		–	Intravenously every three weeks	29.7%	2.3 months	9.9 months	14%	9.5%	47.6%
KEYNOTE‐021, ‐189, ‐407 (NCT02039674, NCT02578680, NCT02775435)^[^ ^148^ ^]^	Pooled Analysis	Pembrolizumab +chemotherapy	Untreated advanced NSCLC without EGFR and ALK	Chemotherapy	Intravenously every three weeks vs. Intravenously every three weeks	41.0% vs. 13.0%	6.9 months vs. 4.1 months	18.8 months vs. 7.6 months	59.8% vs. 45.3%	25.5% vs. 10.9%	8.6% vs. 24.2%
HARMONI‐2 (NCT05499390)^[^ ^171^ ^]^	Phase III, randomized	AK112	Untreated locally advanced or metastatic PD‐L1+ NSCLC without EGFR and ALK	Pembrolizumab	Intravenously every three weeks vs. Intravenously every three weeks	–	8.0 months vs. 5.0 months	–	29% vs. 16%^*)^	2% vs. 3%^*)^	–
Impower 133 (NCT02763579) ^[^ ^151^ ^]^	Phase III, randomized	Atezolizumab + chemotherapy	Untreated Extensive‐Stage SCLC	Chemotherapy	Intravenously every three weeks vs. Intravenously every three weeks	–	–	12.3 months vs. 10.3 months	58.1% vs. 57.6%(all)	–	–
CASPIAN (NCT03800134) ^[^ ^152^ ^]^	Phase III, randomized	Durvalumab + chemotherapy	Untreated Extensive‐Stage SCLC	Chemotherapy	Intravenously every three weeks vs. Intravenously every three weeks	–	–	11.3 months vs. 8.5 months	62% vs. 62%	9% vs. 9%(all)	–
ASTRUM‐005 (NCT04063163)^[^ ^154^ ^]^	Phase III, randomized	Serplulima + chemotherapy	Untreated Extensive‐Stage SCLC	Chemotherapy	Intravenously every three weeks vs. Intravenously every three weeks	–	–	15.4 months vs. 10.9 months	33.2% vs. 27.6%(all)	8.0% vs. 7.7%(all)	–
C‐Brain (NCT04291092)^[^ ^150^ ^]^	Phase II	SRS or WBRT + Camrelizumab + chemotherapy	Untreated advanced NSCLC	–	Intravenously every three weeks	78.5%	6‐month PFS of 71·7%	–	55%	–	2%
**Antibody‐Drug Conjugates**											
**HER‐2**											
DESTINY‐Lung01, DESTINY‐Lung02 (NCT03505710, NCT04644237)^[^ ^155^ ^]^	Phase II	T‐DXd	HER2‐mutant NSCLC	–	Intravenously every three weeks	50.0%	–	–	38.2%^*)^	–	–
**HER‐3**											
HERTHENA‐Lung01 (NCT04619004)^[^ ^172^ ^]^	Phase II	HER3‐DXd	Treated with EGFR‐TKIs and chemotherapy NSCLC	–	Intravenously every three weeks	33.3%	–	–	64.9% ^*)^	7.1%^*)^	13.3%
**DLL3**											
TAHOE (NCT03061812) ^[^ ^157^ ^]^	Phase III, randomized	Rova‐T	DLL3‐high advanced or metastatic SCLC	Topotecan	Intravenously every six weeks vs. Intravenously every six weeks	–	–	7.3 months vs. 8.8 months	64% vs. 88%^*)^	–	–
MERU (NCT03033511)^[^ ^158^ ^]^	Phase III, randomized	Rova‐T	Patients without disease progression after platinum‐based chemotherapy of extensive‐stage‐SCLC	Placebo	Intravenously every six weeks vs. Intravenously every six weeks	–	–	10.2 months vs. 10.7 months	59% vs. 30% ^*)^	20% vs. 7%^*)^	–
**TROP2**											
TROPION‐Lung 05 (NCT04484142) ^[^ ^159^ ^]^	Phase II	Dato‐DXd	Treated advanced/metastatic NSCLC	–	Intravenously every three weeks	22.0%	–	–	47.4%^*)^	9.5%^*)^	27%

*) Data were derived from analysis of the entire patient cohort without subgroup analysis. BM, brain metastases; SRS, stereotactic radiosurgery; WBRT, whole‐brain radiation therapy.

Despite advances with TKIs and immune checkpoint inhibitors in managing BM, BBB efflux mechanisms and secondary drug resistance continue to limit efficacy. Repurposing existing drugs and developing novel therapeutic agents, including dual‐targeting compounds and nanoparticle‐based formulations, offer promising advancements in the treatment of BM.

### 6.4 Dual‐Targeting Agents

AK112 is a novel bispecific antibody targeting PD‐1 and VEGF. In the presence of VEGF, AK112 forms soluble complexes with VEGF dimers, enhancing PD‐1 binding avidity and PD‐1/PD‐L1 blockade. PD‐1 engagement likewise improves VEGF binding affinity, amplifying VEGF signaling inhibition. This reciprocal mechanism drives synergistic dual‐target activity. AK112 is a novel bispecific antibody targeting PD‐1 and VEGF. In the HARMONI‐A study,^[^ ^160^ ^]^ ivonescimab plus chemotherapy significantly prolonged PFS compared with chemotherapy alone in patients with BM, with a median PFS of 5.75 versus 4.14 months (HR = 0.40, 95% CI 0.22–0.73). The HARMONI‐2 study^[^ ^161^ ^]^ compared AK112 with pembrolizumab as first‐line treatments for locally advanced or metastatic NSCLC in patients with PD‐L1–positive tumors. AK112 significantly improved PFS in the overall population (HR = 0.51, 95% CI 0.384–0.69) and showed a trend toward improved PFS in patients with BM (HR = 0.55, 95% CI 0.28–1.05) compared to pembrolizumab. MET amplification is a well‐characterized resistance driver to EGFR‐TKIs. Amivantamab, a bispecific antibody targeting EGFR and MET, combined with lazertinib, demonstrated clinically meaningful improvements in iPFS in NSCLC. iPFS was 25.4 months with amivantamab plus lazertinib versus 22.2 months with osimertinib (HR = 0.79, 95% CI 0.61–1.02; p = 0.07), with 3‐year iPFS rates of 36% versus 18%, respectively.

Yin et al.^[^ ^162^ ^]^ developed a co‐delivery system for simvastatin and gefitinib, engineered with an anti‐PD‐L1 nanobody and a TfR‐binding peptide T12. This system overcame EGFRT790M‐associated TKI resistance in NSCLC with BM by penetrating the BBB, repolarizing tumor‐associated macrophages and suppressing EGFR/Akt/Erk signaling through ROS elevation. Fu et al.^[^ ^163^ ^]^ showed that EGFR‐TKI therapy upregulates CTLA‐4 expression in T cells, promoting an immunosuppressive microenvironment. Combining CTLA‐4 blockade with EGFR‐TKIs enhanced the efficacy of EGFR‐TKIs, either alone or in combination with PD‐1 inhibitors (Table 2).

### 6.5 Nanoparticle Delivery

The BBB limits drug delivery to the brain. Inspired by the invasive capacity of *Escherichia coli* K1 (EC‐K1) in bacterial meningitis, Chen et al.^[^ ^164^ ^]^ developed biomimetic self‐assembled nanoparticles using a lipopolysaccharide‐free EC‐K1 outer membrane to facilitate brain‐targeted delivery. These nanoparticles exhibited prolonged circulation, enhanced intracranial distribution, and excellent biocompatibility. Fu et al.^[^ ^165^ ^]^ developed an inhalable nanoliposome platform for the co‐delivery of osimertinib and a plasmid encoding siRNA. Mimicking natural pulmonary surfactant, these nanoparticles traversed pulmonary barriers, accumulated in lung tissues, and induced exosome‐mediated delivery of therapeutic cargo to the brain.^[^ ^165^ ^]^ This dual‐function system inhibited primary and metastatic tumors and activated anti‐tumor immunity. This nanoparticle delivery system penetrates the BBB, providing new insights for future research to overcome BBB restraint. Jiang et al.^[^ ^166^ ^]^ identified LPCAT1 as specifically upregulated in BM compared with primary lung tumors. They engineered HEK293T‐derived exosomes with EGFR‐targeted single‐chain antibody fragments (scFv) to create a precision delivery system (exo‐scFv) to achieve EGFR‐specific targeting and enhanced BBB penetrability. Loading these exosomes with LPCAT1 siRNA (siLPCAT1) enabled successful BBB crossing and siRNA delivery to BM, resulting in significant tumor inhibition in vivo, without notable toxicity.

## 7 New Therapeutic Targets

Advances in bioinformatics have greatly enhanced our understanding of the brain's distinct microenvironment, uncovering several promising therapeutic targets within the TME of BM. This section synthesizes emerging microenvironment‐specific targets and their interaction mechanisms, offering novel translational opportunities for the treatment of BM.

### 7.1 Targeting BBB and BM Vasculature

The BBB is a key defense against tumor infiltration. Tumor cells exploit several mechanisms to breach the BBB. For example, they secrete cathepsin S to cleave JAM‐B and upregulate PTGS2, HB‐EGF, ST6GALNAC5,^[^ ^173^ ^]^ PLGF,^[^ ^174^ ^]^ and PLEKHA5,^[^ ^175^ ^]^ all of which increase BBB permeability. Karreman et al.^[^ ^176^ ^]^ demonstrated that during early metastasis, matrix metalloproteinase 9 (MMP9) is secreted, causing localized endothelial barrier disruption. After penetrating the BBB and vascular basement membrane, MMP9 stimulates capillary remodeling and promotes micrometastasis formation. Chang et al.^[^ ^177^ ^]^ reported that YTHDF3 overexpression in patients with breast cancer was associated with BM and enhanced translation of m6A‐enriched transcripts (ST6GALNAC5, GJA1, EGFR). It also promoted tumor cell, brain endothelial cell, and astrocyte interactions; BBB leakage; and angiogenesis. YTHDF3 knockdown did not affect primary tumor growth but impaired BM formation and improved survival in murine models.

Boire et al.^[^ ^178^ ^]^ discovered that complement component 3 (C3), secreted by tumor cells, binds to C3a receptors on the choroid plexus, disrupting the blood‐CSF barrier. This disruption permits plasma‐derived growth factors to enter the CSF and drive leptomeningeal metastasis. Targeting C3aR effectively suppressed leptomeningeal metastasis in breast and lung cancer models. Whiteley et al.^[^ ^179^ ^]^ described a BBB‐independent metastatic route. Tumor cells expressing integrin α6 bind to laminin in the vascular basement membrane and migrate along laminin‐rich perivenous spaces into the leptomeninges, where they interact with perivascular meningeal macrophages to induce glial‐derived neurotrophic factor (GDNF), facilitating tumor growth in the leptomeninges. Intrathecal GDNF blockade, macrophage‐specific GDNF ablation, or deletion of NCAM (the GDNF receptor) inhibited leptomeningeal tumor growth.

CD276, an immune checkpoint molecule, was significantly upregulated in the BM vasculature of breast, lung, and melanoma models. Anti‐CD276 antibodies extended survival and increased infiltration of CD3^+^CD8^+^ T cells, CD44^+^CD62L^+^ central memory, and CD44^−^CD62L^+^ naive CD8^+^ T cells, as well as GZMB^+^CD8^+^ T cells.^[^ ^180^ ^]^

### 7.2 Targeting Astrocytes

Astrocytes secrete plasminogen activators to suppress metastasis and invasion; however, tumor‐derived serpins counteract this effect.^[^ ^85^ ^]^ Chen et al.^[^ ^181^ ^]^ identified CX43‐ and PCDH7‐mediated gap junctions between astrocytes and metastatic cells that enable cGAMP transfer and STING pathway activation. This process stimulates astrocyte cytokine release (IFN‐α and TNF), activating STAT1 and NF‐κB pathways in cancer cells and promoting BM. Inhibiting these gap junction‐mediated processes suppresses the progression of metastatic lesions.

Qu et al.^[^ ^120^ ^]^ found that SCLC cells secrete the brain development factor reelin, which binds with astrocytic VLDLR, promoting astrocyte migration to BM following intracranial injection into mouse brains. Astrocytes then secrete SERPINE1, supporting SCLC proliferation. Priego et al.,^[^ ^182^ ^]^ using single‐cell RNA sequencing, identified STAT3^+^ astrocytes that produce TIMP1. This binds CD63 on CD8^+^T cells, inhibiting their activation and forming an immune heterogeneous microenvironment, thereby promoting BM growth.

Tang et al.^[^ ^183^ ^]^ reported that reactive astrocytes secrete interleukin 11 (IL‐11) to promote PD‐L1 expression, resulting in the immune escape of BM from EGFR‐mutated NSCLC by increasing CD8+ T lymphocyte apoptosis. Additionally, IL‐11 promoted immune escape by binding to its intrinsic receptor component glycoprotein 130 (gp130). Targeting both gp130 and EGFR inhibits BM growth and prolongs survival in mice.

### 7.3 Targeting Microglia

Microglia act as CNS‐resident macrophages that initially help repair BBB damage caused by CTCs and facilitate the formation of new BM after systemic treatment.^[^ ^83^ ^]^ Jin et al.^[^ ^184^ ^]^ showed that in A549‐derived lung cancer BM models, tumor‐derived IL6 activated the JAK2/STAT3 pathway, inducing the polarization of microglia from M1 (classical macrophages) to M2 (alternatively activated macrophages), supporting BM formation. Inhibiting this axis significantly reduced BM incidence. Wang et al.^[^ ^185^ ^]^ discovered that HSP47 was significantly upregulated in breast and lung BM, promoting type I collagen deposition and activating the integrin α2β1/NF‐κB pathway. This further enhanced M2 polarization and suppressed CD8^+^ T‐cell immune responses, thus facilitating BM. Blocking HSP47‐collagen interactions restored anti‐tumor immunity and improved the response to anti‐PD‐L1 immunotherapy.

CXCL10, an immune modulator highly expressed in BM, recruits V‐domain Ig suppressor of T cell activation–high (VISTA–high) PD‐L1^+^ microglia to BM lesions, further dampening T‐cell function. Co‐treatment with αVISTA and αPD‐L1 antibodies reduced tumor burden and improved T‐cell activation, suggesting a potential therapeutic strategy for enhancing anti‐tumor immunity in BM.^[^ ^93^ ^]^

### 7.4 Targeting Metastasis‐Promoting “Messengers”

Lipocalin‐2 (LCN2)—a secretory protein transporting various lipophilic molecules such as steroids, lipopolysaccharides, iron, and fatty acids—is overexpressed in cancer cells within the CSF of patients with leptomeningeal metastasis from breast or lung cancer. Chi et al.^[^ ^186^ ^]^ linked high LCN2 levels to poor patient survival and showed that cancer cells compete with macrophages for extracellular iron in the CSF, promoting M2 polarization and immune suppression. Notably, iron chelation significantly improved survival in leptomeningeal metastasis models. Adler et al.^[^ ^89^ ^]^ found that granulocyte‐derived LCN2 activated astrocytes via SCL22A17, leading to myeloid recruitment and inflammatory microenvironment formation in melanoma and breast cancer BM. Mice transplanted with Lcn2−/− bone marrow had significantly reduced BM burden.

**Figure 3:**
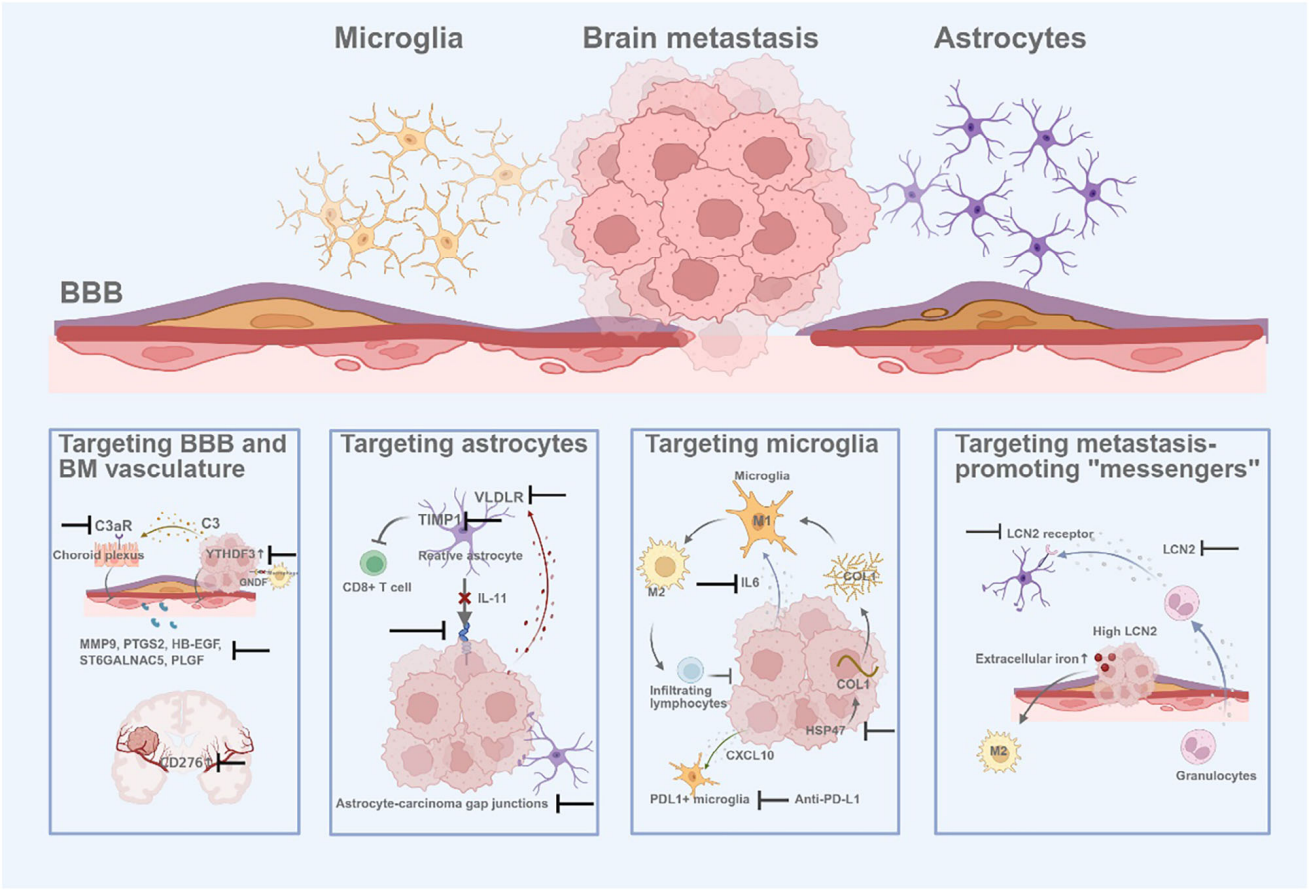
New targets in the BM microenvironment. This figure illustrates emerging therapeutic targets within the BM microenvironment, including those associated with the BBB (C3, C3aR, MMP9, PTGS2, HB‐EGF, ST6GALNAC5, PLGF, and CD276), astrocytes (VLDLR, TIMP1, IL‐11, astrocyte‐carcinoma gap junctions), microglia (IL‐6, HSP47, and PD‐L1), and metastasis‐promoting mediators (LCN2 and its receptor). These targets represent potential avenues for therapeutic intervention by modulating the unique cellular and molecular components of the BM microenvironment. BM: brain metastases, BBB: blood–brain barrier.

Parida et al.^[^ ^187^ ^]^ analyzed metabolic differences in various breast cancer. Synchronous BM preferentially utilized glucose, whereas metachronous and occult BM relied on glutamine. Unlike the former, occult BM secreted minimal lactate. Lactate produced by synchronous and metachronous metastases impaired NK cell cytotoxicity, facilitating immune evasion and metastatic outgrowth. Consequently, targeting lactate metabolism reduced the BM burden (**Figure** **3**).

## 8 Summary

BM, a severe complication of lung cancer, is associated with poor prognosis and represents a leading cause of death. Approximately 40% of patients with NSCLC and 40–50% of those with SCLC develop BM, contributing to substantial morbidity and mortality.

Molecular subtypes exhibit distinct BM characteristics and greater heterogeneity than primary tumors. Understanding the prognostic significance of molecular subtypes and other clinical factors is crucial for guiding precision therapy and identifying responsive patient populations. In addition, using AI in medical imaging and liquid biopsy continues to show promise in oncology, enabling tumor detection, prognostic evaluation, and monitoring of minimal residual disease and recurrence. Preclinical research has also advanced, particularly through the development of animal and organoid models, which have facilitated the progress in both mechanistic studies and personalized therapeutic approaches. Nevertheless, prognosis remains poor, and the underlying molecular and cellular mechanisms of BM have not been fully elucidated. Key unresolved questions include identifying the molecular drivers and CTCs involved in BM, elucidating their roles in metastasis, and determining their potential as predictive or therapeutic targets.

Therefore, a comprehensive understanding of BM pathogenesis, coupled with the development of targeted therapeutic interventions based on novel molecular findings, is critical for improving outcomes in patients with lung cancer‐associated BM.

## Conflict of Interest

The authors declare no conflict of interest.

## Data Availability

I agree that if accepted, the article will be published open access and that the Corresponding Author is responsible for arranging payment of the APC.
